# Metabolic and Epigenetic Mechanisms in Hepatoblastoma: Insights into Tumor Biology and Therapeutic Targets

**DOI:** 10.3390/genes15111358

**Published:** 2024-10-22

**Authors:** Yuanji Fu, Raquel Francés, Claudia Monge, Christophe Desterke, Agnès Marchio, Pascal Pineau, Yunhua Chang-Marchand, Jorge Mata-Garrido

**Affiliations:** 1 CNRS, INSERM, Institut Necker Enfants Malades, Université Paris Cité, 75015 Paris, France; yuanji.fu@inserm.fr (Y.F.); yunhua.chang-marchand@inserm.fr (Y.C.-M.); 2 Energy & Memory, Brain Plasticity Unit, CNRS, ESPCI Paris, PSL Research University, 75006 Paris, France; raquel.frances@espci.fr; 3 INSERM U993, Unité Organisation Nucléaire et Oncogenèse, Institut Pasteur, Université Paris Cité, 75006 Paris, France; claudia.monge@pasteur.fr (C.M.); agnes.marchio@pasteur.fr (A.M.); pascal.pineau@pasteur.fr (P.P.); 4 Faculté de Médecine du Kremlin Bicêtre, Université Paris-Sud, Université Paris-Saclay, 94270 Le Kremlin-Bicêtre, France; christophe.desterke@inserm.fr

**Keywords:** hepatoblastoma, cancer metabolism, epigenetics, tumor biology, therapeutic targets

## Abstract

Background: Hepatoblastoma, the most common pediatric liver malignancy, is characterized by significant molecular heterogeneity and poor prognosis in advanced stages. Recent studies highlight the importance of metabolic reprogramming and epigenetic dysregulation in hepatoblastoma pathogenesis. This review aims to explore the metabolic alterations and epigenetic mechanisms involved in hepatoblastoma and how these processes contribute to tumor progression and survival. Methods: Relevant literature on metabolic reprogramming, including enhanced glycolysis, mitochondrial dysfunction, and shifts in lipid and amino acid metabolism, as well as epigenetic mechanisms like DNA methylation, histone modifications, and non-coding RNAs, was reviewed. The interplay between these pathways and their potential as therapeutic targets were examined. Results: Hepatoblastoma exhibits metabolic shifts that support tumor growth and survival, alongside epigenetic changes that regulate gene expression and promote tumor progression. These pathways are interconnected, with metabolic changes influencing the epigenetic landscape and vice versa. Conclusions: The dynamic interplay between metabolism and epigenetics in hepatoblastoma offers promising avenues for therapeutic intervention. Future research should focus on integrating metabolic and epigenetic therapies to improve patient outcomes, addressing current gaps in knowledge to develop more effective treatments.

## 1. Introduction

Hepatoblastoma (HB), the most common pediatric liver cancer, is driven by complex molecular mechanisms that promote tumorigenesis and therapeutic resistance. Recent research has highlighted the critical roles of metabolic reprogramming and epigenetic dysregulation in HB biology. This paper aims to provide insights into the interplay between these mechanisms, focusing on their contribution to tumor progression and potential therapeutic targeting.

The specific objectives of this review are to (1) analyze the key metabolic pathways altered in HB, (2) explore epigenetic modifications contributing to its malignancy, and (3) evaluate emerging therapeutic strategies targeting these processes. By consolidating recent findings, the goal is to identify novel therapeutic approaches for improving HB treatment outcomes.

This review targets researchers, clinicians, and oncologists working on pediatric liver cancer, with an emphasis on advancing precision medicine approaches in hepatoblastoma.

## 2. Overview of Hepatoblastoma

### 2.1. Epidemiology and Incidence

Hepatoblastoma is the most common primary liver malignancy in children, accounting for approximately 1% of all pediatric cancers. It predominantly affects infants and young children, with the majority of cases diagnosed before the age of 3 [1,2]. The incidence of hepatoblastoma has been gradually increasing in recent decades, with current rates ranging from 1.5 to 2.5 cases per million children worldwide [1,2]. Notably, the incidence is higher in premature infants and in certain geographic regions, including North America, Asia and Europe [3]. While the reasons for this rising trend are not entirely understood, improvements in diagnostic techniques and increased survival rates of premature infants have been proposed as contributing factors.

### 2.2. Etiology and Risk Factors

The etiology of hepatoblastoma remains largely unknown, but several genetic and environmental factors have been implicated. Key risk factors include (Table 1):

Other possible environmental risk factors include prenatal exposure to pesticides, smoking, alcohol, and specific carcinogens, though these associations are less well defined [10,11,12,13].

### 2.3. Pathogenesis

Hepatoblastoma originates from the transformation of undifferentiated hepatoblasts, embryonic liver progenitor cells, with its molecular pathogenesis involving a complex interplay of genetic and epigenetic alterations [13,14]. Central to these are disruptions in the Wnt/β-catenin and Hepatocyte Growth Factor (HGF)/c-Met pathways [15]. Mutations in the CTNNB1 gene, which encodes β-catenin, are present in approximately 70–80% of hepatoblastoma cases and result in aberrant activation of the Wnt signaling pathway, leading to uncontrolled cell proliferation and tumor development [15]. Moreover, dysregulation of the HGF/c-Met signaling axis, including overexpression of the c-Met receptor, plays a crucial role in hepatoblastoma oncogenesis by promoting cellular proliferation, migration, and survival [16]. Other molecular abnormalities contributing to hepatoblastoma development include overexpression of MYC [17] and IGF2 [18], as well as various epigenetic modifications, such as abnormal DNA methylation patterns and histone modifications. These will be discussed in more detail in subsequent sections.

### 2.4. Clinical and Histological Classification

Hepatoblastoma is a highly heterogeneous tumor that presents in several histological subtypes. It is primarily classified into two types: epithelial and mixed epithelial-mesenchymal [19,20]. The epithelial type consists of cells that resemble normal fetal or embryonic liver tissue and is further subdivided into two subtypes: the fetal subtype, characterized by cells similar to early fetal hepatocytes and generally associated with a more favorable prognosis, and the embryonal subtype, which comprises less differentiated, more aggressive cells resembling early liver progenitors [19,20]. The mixed epithelial-mesenchymal type contains both epithelial and mesenchymal components, such as osteoid, cartilage, or muscle. The presence of mesenchymal elements, particularly osteoid production, is linked to a more aggressive clinical course [19,20]. Recent molecular profiling studies have introduced additional classification schemes based on genetic and epigenetic alterations. This molecular heterogeneity underscores the need for personalized treatment approaches tailored to the tumor’s genetic profile [14,21].

### 2.5. Diagnosis

The diagnosis of hepatoblastoma relies on a combination of clinical presentation, imaging, laboratory tests, and histopathological examination [22,23]. Initial imaging typically begins with ultrasound to detect liver masses, followed by more detailed contrast-enhanced CT or MRI to assess the tumor’s size, extent, and vascular involvement. A liver biopsy is crucial for confirming the diagnosis through histological examination and determining the tumor subtype, with immunohistochemistry for markers like β-catenin and AFP providing further diagnostic support [24,25]. Elevated serum α-fetoprotein (AFP) is a key marker for hepatoblastoma, although it may be normal in certain subtypes, such as small, well-differentiated fetal tumors. AFP levels are also valuable for tracking treatment response and detecting recurrence [24,25].

### 2.6. Staging

Staging is critical for treatment planning and prognosis in hepatoblastoma. The most commonly used staging system is the PRETEXT (PRE-treatment EXTent of disease) system, which classifies tumors based on the extent of liver involvement and the presence of extrahepatic disease [26]. The PRETEXT system divides the liver into four sections, and tumors are categorized based on how many sections are involved:

PRETEXT I: One section involved,
PRETEXT II: Two sections involved,
PRETEXT III: Three sections involved, and
PRETEXT IV: All four sections involved.

Additional factors, such as vascular invasion, extrahepatic disease, and metastasis, are also considered in the final-stage assignment.

### 2.7. Prognosis and Survival

The prognosis of hepatoblastoma depends on factors such as tumor stage, histology, and response to therapy. Survival rates have improved significantly, with localized disease achieving survival rates of over 70–80%. However, outcomes remain poor for patients with advanced or metastatic disease, with 5-year survival rates ranging from 30–50% [27,28]. Tumor histology also influences prognosis, with the fetal subtype being associated with better outcomes, while the embryonal and mixed types are linked to more aggressive disease. Complete surgical resection remains a key predictor of survival, and tumors that are initially inoperable often require neoadjuvant chemotherapy to shrink them before surgery [27,28]. Although hepatoblastoma is generally chemosensitive—standard multi-agent regimens include cisplatin, doxorubicin, and vincristine—patients who respond poorly to chemotherapy tend to have worse outcomes [27,28].

### 2.8. Current Treatment Strategies

The treatment of hepatoblastoma involves a multimodal approach, combining surgery, chemotherapy, and, in some cases, liver transplantation. The treatment plan is tailored to the tumor’s stage, size, and location [25,26]. Complete surgical resection is the cornerstone of treatment and is curative in most localized cases, while unresectable tumors often require neoadjuvant chemotherapy to shrink the tumor and enable resection. Standard chemotherapy protocols, such as those from SIOPEL and COG, are cisplatin-based, with more intensive regimens used for high-risk patients. In cases where tumors are unresectable or involve all liver segments, liver transplantation offers the best chance for long-term survival [25,26]. Additionally, emerging therapies targeting specific molecular pathways, such as Wnt/β-catenin inhibitors, are under investigation, and immunotherapy is also being explored, although it remains in early stages of development [25,26].

### 2.9. Challenges and Future Directions

Despite advances in treatment, several challenges remain in the management of hepatoblastoma, particularly for patients with advanced disease or relapse. Key challenges include tumor heterogeneity, characterized by the molecular and histological diversity of hepatoblastoma, which complicates the personalization of treatment; drug resistance, where some tumors develop resistance to standard chemotherapy, highlighting the need for new therapeutic strategies; and long-term health complications, such as hearing loss (due to cisplatin), cardiotoxicity, and secondary malignancies [29].

Ongoing research into the molecular drivers of hepatoblastoma, particularly in the areas of metabolism and epigenetics, is expected to pave the way for novel therapeutic approaches that target the unique vulnerabilities of these tumors, potentially improving outcomes for patients with this challenging disease [30,31].

This review aims to provide a comprehensive and detailed examination of the metabolic and epigenetic underpinnings of hepatoblastoma, focusing on how these factors contribute to the tumor’s pathogenesis, progression, and therapeutic responses. We will emphasize two interconnected aspects: metabolic reprogramming and epigenetic dysregulation in this disease. The current understanding of how these metabolic and epigenetic alterations contribute to the initiation, maintenance, and progression of hepatoblastoma will be highlighted. Furthermore, we will explore the interplay between metabolic changes and epigenetic regulation, underscoring how this crosstalk influences tumor behavior and therapeutic resistance. By integrating knowledge from both fields, this review seeks to provide new insights into potential therapeutic strategies targeting these critical pathways, ultimately aiming to identify opportunities for more effective, personalized treatments.

## 3. Metabolism in Hepatoblastoma

**Figure 1:**
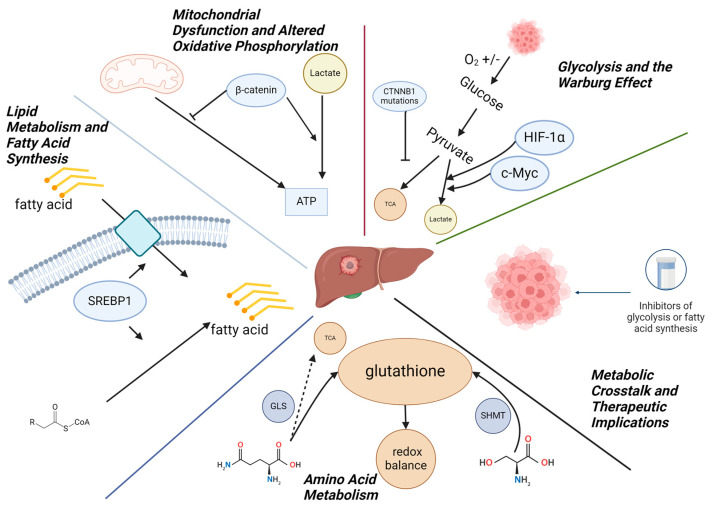
Summary of metabolic alterations in hepatoblastoma. The figure shows the main metabolic pathways altered in hepatoblastoma, including mitochondrial alterations, predilection for glycolysis associated with the Warburg effect, and disturbances in amino acid and lipid metabolism. Taken together, these characteristics may have important implications for the establishment of a possible treatment.

Metabolic reprogramming is a hallmark of cancer, and hepatoblastoma is no exception. Like many malignancies, hepatoblastoma cells undergo profound metabolic changes to support their rapid proliferation and survival in a nutrient-limited environment. These changes allow tumor cells to optimize energy production, macromolecule biosynthesis, and redox balance. In hepatoblastoma, several metabolic pathways are dysregulated, including glycolysis, mitochondrial metabolism, lipid metabolism, and amino acid metabolism [30,31]. This section provides a detailed overview of the metabolic alterations in hepatoblastoma, with a focus on the key molecular pathways and genes that drive these changes (Figure 1).

### 3.1. Glycolysis and the Warburg Effect

One of the most significant metabolic alterations in hepatoblastoma is the upregulation of glycolysis, even in the presence of sufficient oxygen—a phenomenon known as the Warburg effect [32,33]. Normally, hepatocytes rely on oxidative phosphorylation (OXPHOS) in the mitochondria for ATP production. However, hepatoblastoma cells switch to aerobic glycolysis, which, although less efficient in generating ATP, provides essential building blocks such as nucleotides, amino acids, and lipids required for rapid cell division [32,33]. Key molecular drivers of the Warburg effect in hepatoblastoma include Hypoxia-Inducible Factor 1-α (HIF-1α), which, stabilized by hypoxia or Wnt/β-catenin signaling, promotes glycolysis by upregulating enzymes like hexokinase 2 (HK2), phosphofructokinase (PFK1), and pyruvate kinase M2 (PKM2). HIF-1α also enhances lactate dehydrogenase A (LDHA), redirecting pyruvate towards lactate production [32,33]. Additionally, c-Myc, which is overexpressed in hepatoblastoma, accelerates glycolysis by upregulating glucose transporters (GLUT1, GLUT3) and enzymes like LDHA, while also diverting glycolytic intermediates to the pentose phosphate pathway (PPP) for nucleotide synthesis [32,33]. Finally, mutations in CTNNB1, which encodes β-catenin and is found in 70–80% of hepatoblastoma cases, further drive metabolic reprogramming by increasing the expression of pyruvate dehydrogenase kinase 1 (PDK1). This enzyme blocks the entry of pyruvate into the TCA cycle, promoting lactate production and enhancing cell survival in hypoxic conditions [30,31,32,33].

### 3.2. Mitochondrial Dysfunction and Altered Oxidative Phosphorylation

While glycolysis is upregulated in hepatoblastoma, the role of OXPHOS varies depending on tumor subtype [34]. Some hepatoblastoma cells, particularly in well-differentiated fetal-type tumors, continue to rely on mitochondria for energy production, whereas more aggressive embryonal-type tumors often exhibit mitochondrial dysfunction [34]. One key alteration is the inhibition of pyruvate entry into the TCA cycle by pyruvate dehydrogenase kinase 1 (PDK1), which is upregulated by β-catenin and phosphorylates pyruvate dehydrogenase (PDH), shifting metabolism from mitochondrial ATP production to lactate generation [35,36]. Mitochondrial biogenesis, regulated by factors like PGC1α, may also be altered, while dysregulation of mitochondrial dynamics, including fission and fusion processes, can impact the balance between OXPHOS and glycolysis [32,34,35]. Furthermore, mitochondrial dysfunction in hepatoblastoma often leads to increased reactive oxygen species (ROS) production, which promotes genomic instability and activates pro-survival pathways, such as the NRF2 antioxidant response [14,36]. NRF2 upregulates detoxifying enzymes like glutathione peroxidase (GPx) and superoxide dismutase (SOD), helping tumor cells survive oxidative stress [14,36].

### 3.3. Lipid Metabolism and Fatty Acid Synthesis

Lipid metabolism plays a crucial role in the survival and growth of hepatoblastoma cells, which require large amounts of lipids for membrane synthesis, energy storage, and signaling. To meet these demands, hepatoblastoma cells enhance both de novo fatty acid synthesis and lipid uptake. A key regulator in this process is Sterol Regulatory Element-Binding Protein 1 (SREBP1), which upregulates enzymes such as acetyl-CoA carboxylase (ACC) and fatty acid synthase (FASN) to drive the conversion of acetyl-CoA into long-chain fatty acids, essential for membrane production in rapidly proliferating cells [37,38]. Additionally, some hepatoblastoma cells utilize fatty acid oxidation (FAO) to generate ATP, with the enzyme carnitine palmitoyltransferase 1 (CPT1) facilitating the transport of fatty acids into mitochondria for β-oxidation [39]. This metabolic flexibility enables the tumor to switch between lipid synthesis and breakdown depending on nutrient availability. Hepatoblastoma cells also accumulate lipid droplets, which store triacylglycerols and cholesteryl esters, serving as an energy reservoir that can be mobilized during periods of metabolic stress.

### 3.4. Amino Acid Metabolism

Amino acid metabolism is critical for meeting the biosynthetic and redox demands of hepatoblastoma cells, with glutamine and serine playing particularly central roles. Through the process of glutaminolysis, glutamine provides nitrogen and carbon for proliferating cells. It is converted to glutamate and then to α-ketoglutarate, which fuels the TCA cycle to sustain energy production when glucose is directed toward biosynthesis [40]. The enzyme glutaminase (GLS), often upregulated in hepatoblastoma, catalyzes the conversion of glutamine to glutamate. This glutamate can either be used to produce glutathione, maintaining redox balance, or to replenish TCA cycle intermediates [41]. Similarly, serine metabolism is crucial as it supports nucleotide and lipid synthesis by being produced from the glycolytic intermediate 3-phosphoglycerate via phosphoglycerate dehydrogenase (PHGDH), another enzyme frequently upregulated in hepatoblastoma [42]. Serine also contributes to the one-carbon cycle, generating one-carbon units essential for nucleotide synthesis and methylation reactions. Additionally, glycine, produced from serine-by-serine hydroxymethyltransferase (SHMT), aids in purine synthesis and glutathione production, further supporting cellular proliferation and redox balance [42].

### 3.5. Metabolic Crosstalk and Therapeutic Implications

Metabolic pathways in hepatoblastoma are not isolated; they interact in a highly coordinated manner to sustain tumor growth. For example, the glycolytic shift in hepatoblastoma not only provides energy but also feeds biosynthetic pathways such as the pentose phosphate pathway (PPP), which produces NADPH and ribose-5-phosphate for nucleotide synthesis [42]. Similarly, lipid metabolism interacts with amino acid metabolism, as acetyl-CoA produced from fatty acid oxidation can enter the TCA cycle or be used for histone acetylation, linking metabolism to epigenetic regulation [39,40,43].

Targeting these metabolic pathways holds significant therapeutic potential. Inhibitors of glycolysis (e.g., 2-deoxyglucose) or fatty acid synthesis (e.g., FASN inhibitors) are being explored as potential therapies for hepatoblastoma [44,45]. Understanding the metabolic flexibility and redundancy in these pathways is crucial for developing effective combination therapies that target multiple aspects of hepatoblastoma metabolism.

## 4. Epigenetic Regulation in Hepatoblastoma

**Figure 2:**
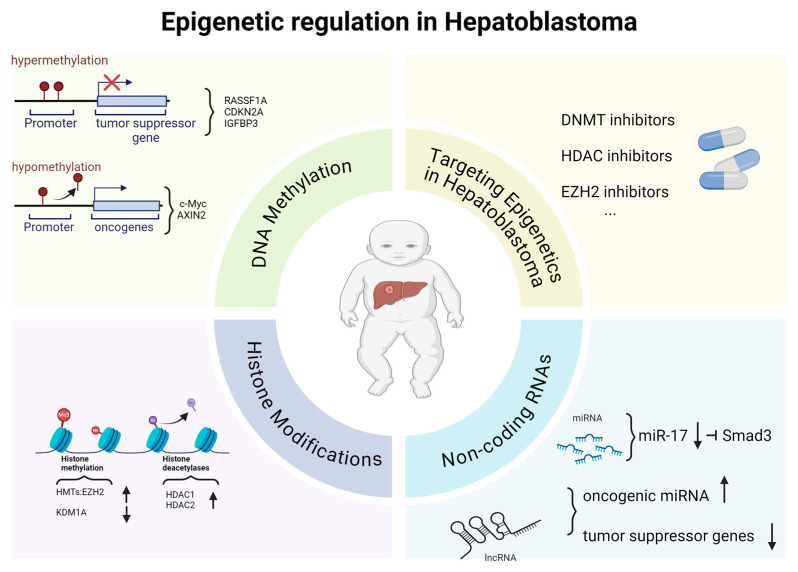
Overview of epigenetic regulation in hepatoblastoma. Regarding DNA methylation, hypermethylation at the promoters of tumor suppressor genes, such as RASSF1A, CDKN2A and IGFBP3, results in their silencing, favoring tumor proliferation. In contrast, hypomethylation in promoters of oncogenes, such as c-MyC and AXIN2, leads to their activation, promoting oncogenesis. Histone modifications also play a crucial role; aberrant histone methylation mediated by HMTs such as EZH2 or demethylases such as KDM1A can favor an oncogenic environment, while excessive deacetylation, facilitated by HDAC1 and HDAC2, represses transcription of key genes in cell regulation. Non-coding RNAs, such as miRNAs, are also altered: the reduction of miR-17 decreases Smad3 inhibition, while the increase in oncogenic lncRNAs and the reduction of those regulating tumor suppressor genes amplify oncogenic effects. These epigenetic alterations represent promising therapeutic targets, with inhibitors of DNMTs, HDACs and EZH2 emerging as potential strategies to reverse these malignant changes and improve outcomes in patients with hepatoblastoma.

Epigenetic regulation plays a central role in the pathogenesis and progression of hepatoblastoma. Unlike genetic mutations that alter the DNA sequence, epigenetic changes modify gene expression without changing the underlying DNA code [46]. These changes include DNA methylation, histone modifications, and the involvement of non-coding RNAs, all of which contribute to the dynamic control of the genome [46]. In hepatoblastoma, epigenetic dysregulation has been shown to influence oncogene activation, tumor suppressor gene silencing, and metabolic reprogramming [47,48]. This section provides a detailed analysis of the key molecular pathways and genes involved in the epigenetic regulation of hepatoblastoma (Figure 2).

### 4.1. DNA Methylation

DNA methylation is a well-characterized epigenetic modification in hepatoblastoma, involving the addition of a methyl group to cytosine in CpG dinucleotides. This modification typically leads to transcriptional repression when it occurs in promoter regions. Abnormal DNA methylation patterns in hepatoblastoma include hypermethylation of tumor suppressor gene promoters and hypomethylation of oncogenes, both of which contribute to tumor initiation and progression [49,50]. For instance, hypermethylation of the RASSF1A promoter, a gene involved in cell cycle regulation and apoptosis, silences its expression in up to 80% of cases, resulting in unchecked cell proliferation [51]. Similarly, the CDKN2A (p16/INK4a) gene, which inhibits cell cycle progression, is often hypermethylated, thereby promoting tumor growth [52]. Furthermore, hypermethylation of IGFBP3, a regulator of insulin-like growth factor activity, removes its inhibitory effect on cell proliferation [53]. Conversely, global hypomethylation can activate oncogenes such as c-Myc, a key driver of cell proliferation and metabolism, leading to more aggressive tumor behavior [54]. Hypomethylation also affects genes in the Wnt/β-catenin pathway, such as AXIN2, further enhancing this crucial tumor-promoting pathway, which is frequently activated through both genetic mutations and epigenetic changes in hepatoblastoma [55].

### 4.2. Histone Modifications

Histone modifications, including methylation and acetylation, play a critical role in regulating gene expression by altering chromatin structure, and their dysregulation in hepatoblastoma significantly contributes to tumorigenesis [48,54]. Histone methyltransferases (HMTs), such as EZH2, which is overexpressed in hepatoblastoma, add repressive marks like H3K27 trimethylation (H3K27me3) to silence tumor suppressor genes, promoting uncontrolled cell proliferation [56]. Conversely, histone demethylases such as KDM1A can remove these repressive marks, thereby activating genes related to cell differentiation and cell cycle regulation. However, their dysregulation can contribute to the undifferentiated state of tumor cells [57]. Histone acetylation, typically associated with gene activation, is also disrupted in hepatoblastoma due to an imbalance between histone acetyltransferases (HATs) and histone deacetylases (HDACs). Overexpression of HDACs, such as HDAC1 and HDAC2, results in the deacetylation of histones at tumor suppressor gene promoters, further repressing their expression and fostering tumor growth [58,59]. HDAC inhibitors, such as vorinostat, have shown promise in preclinical models by restoring acetylation levels and reactivating silenced tumor suppressor genes, although their clinical efficacy remains under investigation [60].

### 4.3. Non-Coding RNAs (ncRNAs)

Non-coding RNAs (ncRNAs), including microRNAs (miRNAs) and long non-coding RNAs (lncRNAs), play critical roles in regulating gene expression at both post-transcriptional and epigenetic levels in hepatoblastoma. They can function as either oncogenes or tumor suppressors by targeting key signaling pathways [61,62]. For instance, miR-17 is frequently upregulated in hepatoblastoma [63] and acts as an oncogene by downregulating Smad3 [64]. Restoring miR-17 expression has demonstrated anti-tumor effects in various models [64]. In contrast, tumor-suppressive miRNAs, such as miR-19b, are often downregulated in this disease [63]. Among lncRNAs, CRNDE (Colorectal neoplasia differentially expressed) and OIP5-AS1 (OPA-interacting protein 5 antisense 1) are notable for their roles in promoting tumor growth and metastasis. CRNDE is elevated in hepatoblastoma, and its knockout inhibits tumor growth and angiogenesis in vivo. Additionally, CRNDE reduces proliferation and viability in hepatoblastoma HuH6 cells by activating the mTOR pathway [65]. Moreover, CRNDE modulates VEGFA expression by suppressing miR-203, which is decreased in hepatoblastoma tissues, thereby facilitating tumor angiogenesis and growth [66]. OIP5-AS1, also elevated in hepatoblastoma, is associated with poor prognosis. It enhances ZEB1 expression by downregulating miR-186-5p, promoting hepatoblastoma cell proliferation, metastasis, and epithelial-to-mesenchymal transition (EMT) [67].

### 4.4. Therapeutic Implications of Targeting Epigenetics in Hepatoblastoma

Given the crucial role of epigenetic dysregulation in hepatoblastoma, targeting epigenetic modifiers represents a promising therapeutic strategy. Several classes of drugs, including DNMT inhibitors (e.g., azacytidine), HDAC inhibitors, and EZH2 inhibitors, are under investigation in preclinical models and clinical trials for hepatoblastoma [3,25,26,27,28,48,58]. These therapies aim to restore normal epigenetic patterns, reactivate silenced tumor suppressor genes, and induce differentiation or apoptosis in cancer cells. Combination therapies targeting both epigenetic modifications and metabolic pathways may offer a more effective approach, given the extensive crosstalk between these systems in hepatoblastoma.

In conclusion, epigenetic regulation in hepatoblastoma involves a complex interplay of DNA methylation, histone modifications, and non-coding RNAs, all of which converge to promote oncogenic gene expression and suppress tumor suppressors. Understanding the molecular mechanisms underlying these changes provides valuable insights into potential therapeutic targets for this pediatric liver cancer.

## 5. Integration of Metabolism and Epigenetics in Hepatoblastoma

**Figure 3:**
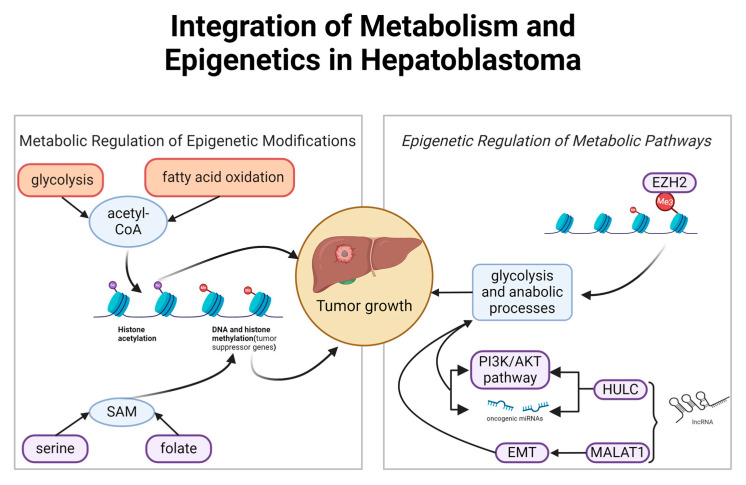
Integrative outline of metabolic and epigenetic regulations in hepatoblastoma. Glycolysis and fatty acid oxidation produce acetyl-CoA, a key molecule for histone acetylation, facilitating the transcription of tumor suppressor genes. In addition, the serine and folate pathway is essential in the generation of SAM (S-adenosylmethionine), the major methyl group donor, vital for DNA and histone methylation, modulating key genes in oncogenesis. Overexpression of oncogenes and silencing of suppressor genes are related to these epigenetic modifications. On the other hand, glycolysis also supplies the anabolic processes necessary for cell proliferation. Overexpression of EZH2, a histone methyltransferase, promotes the repression of suppressor genes, favoring tumor growth. Additionally, activation of the lncRNAs HULC and MALAT1, together with activation of the PI3K/AKT pathway, triggers epithelial-mesenchymal transition (EMT), a key process in metastasis. The integration of these processes in hepatoblastoma reveals a complex circuit in which metabolic changes feed epigenetic alterations, reinforcing tumor proliferation and disease progression. This suggests that simultaneously targeting metabolic and epigenetic pathways could be an effective therapeutic strategy.

The integration of metabolism and epigenetics in hepatoblastoma is a crucial area of study that highlights how metabolic alterations drive epigenetic changes and vice versa, impacting tumor progression and treatment responses. This intricate interplay is evidenced by how metabolites influence epigenetic modifications and how epigenetic changes can, in turn, alter metabolic pathways, creating a dynamic feedback loop essential for tumor development and maintenance (Figure 3).

### Metabolic Regulation of Epigenetic Modifications

Epigenetic modifications, such as histone acetylation and DNA methylation, are intricately linked to metabolic pathways through various metabolites that serve as cofactors or substrates for the enzymes involved in these processes. Histone acetylation, which facilitates gene activation by loosening chromatin structure, is directly regulated by acetyl-CoA, a central metabolite produced through glycolysis and fatty acid oxidation. In hepatoblastoma, increased glycolytic and lipogenic activity raises acetyl-CoA levels, promoting histone acetylation at genes that drive cell proliferation and survival [68,69]. Elevated acetyl-CoA enhances histone acetyltransferase activity, resulting in hyperacetylation that supports tumor growth. Conversely, reduced acetyl-CoA levels due to metabolic stress can lead to histone deacetylation, adversely affecting the expression of genes crucial for tumor adaptation [45]. Similarly, DNA methylation, which typically represses gene expression, is influenced by the one-carbon cycle, which involves metabolites like serine and folate that produce S-adenosylmethionine (SAM), the key methyl donor for DNA and histone methylation [70]. In hepatoblastoma, disturbances in serine metabolism that alter SAM levels can affect the activity of DNA methyltransferases and histone methyltransferases. Increased SAM levels enhance DNA methylation, silencing tumor suppressor genes and fostering malignancy, while decreased SAM levels can lead to hypomethylation, potentially activating oncogenes and contributing to tumorigenesis [70].

## 6. Therapeutic Targets in Hepatoblastoma: Future Directions on Metabolic and Epigenetic Regulation

HB is the most common pediatric liver cancer, and although surgery and chemotherapy are standard treatments, resistance to therapy and recurrence remain significant challenges [1,2,3]. Recent advances in our understanding of HB biology, particularly regarding metabolic reprogramming and epigenetic dysregulation, have opened new avenues for therapeutic intervention (Table 2).

### 6.1. Metabolic Dysregulation in Hepatoblastoma

A prominent feature of hepatoblastoma (HB) metabolism is the upregulation of aerobic glycolysis, commonly known as the Warburg effect [32]. This metabolic shift enables tumor cells to generate ATP even in the presence of oxygen. Key enzymes involved in this process, such as hexokinase 2 (HK2) and pyruvate kinase M2 (PKM2), make attractive therapeutic targets [71,72]. Glycolysis inhibitors, such as 2-deoxy-D-glucose (2-DG), have demonstrated promise in preclinical models by effectively reducing HB cell proliferation (Table 2) [73]. In addition to glycolysis, lipid metabolism is also dysregulated in HB, with increased fatty acid synthesis driven by fatty acid synthase (FASN) contributing to tumor growth (Table 2) [74]. FASN inhibitors, such as orlistat and TVB-2640, have shown potential in preclinical studies by disrupting lipid synthesis and thereby depriving tumor cells of essential components [75,76]. Furthermore, mitochondrial metabolism, particularly oxidative phosphorylation (OXPHOS), is upregulated in some cases of HB (Table 2) [35]. Metformin, which is known for its effects on mitochondrial function, may serve as a potential therapeutic agent for HB due to its ability to inhibit OXPHOS, ultimately leading to reduced tumor growth in preclinical settings (Table 2) [77]. Although clinical data on the use of these metabolic inhibitors in HB remain limited, early preclinical findings suggest that targeting these metabolic pathways could significantly impact tumor progression and provide new treatment options beyond conventional chemotherapy.

### 6.2. Epigenetic Dysregulation in Hepatoblastoma

One of the major epigenetic alterations observed in hepatoblastoma (HB) is the hypermethylation of tumor suppressor genes, such as RASSF1A and CDKN2A, which contributes to their silencing [52,78]. This hypermethylation can be reversed by DNA methyltransferase inhibitors (DNMTis) like 5-azacytidine and decitabine (Table 2), which block the enzymes responsible for adding methyl groups to DNA [79,80,81]. These inhibitors have shown promise in reactivating silenced tumor suppressor genes, sensitizing tumors to chemotherapy, and inhibiting HB cell growth. Another class of epigenetic agents is histone deacetylase inhibitors (HDACis) [82]. HDACs, often overexpressed in HB, promote chromatin condensation and gene silencing. Drugs such as vorinostat and panobinostat inhibit HDACs (Table 2), thereby promoting chromatin relaxation and reactivating silenced genes [81]. In preclinical models, these HDAC inhibitors have demonstrated the ability to reduce tumor growth and induce cell death in HB [58]. Moreover, non-coding RNAs, particularly microRNAs (miRNAs), play a crucial role in the pathogenesis of HB [61,62,63,64]. Dysregulated miRNAs, such as miR-19b, miR-146a, and miR-302d, influence tumor progression by suppressing or enhancing the expression of oncogenes and tumor suppressors (Table 2) [63]. Therapeutic strategies utilizing miRNA mimics or antagomiRs (anti-miRNA agents) are currently being explored to modulate these pathways in preclinical models. While these epigenetic therapies are still in the early stages of clinical development for HB, they show potential in restoring normal gene expression and improving patient outcomes.

### 6.3. Combination Therapies

Given the complexity of hepatoblastoma biology, a multimodal therapeutic approach that targets both metabolic pathways and epigenetic regulators is increasingly being considered. Combination therapies have the potential to overcome resistance to single-agent treatments and enhance overall therapeutic efficacy. For example, the combination of glycolytic inhibitors with histone modifiers has demonstrated synergistic effects in reducing cancer cell viability [83]. Inhibiting glycolysis compromises the tumor’s energy supply, while epigenetic modulators, such as histone modifiers, reprogram the expression of key genes involved in tumor survival. This dual strategy has shown greater tumor suppression in preclinical models compared to single-agent treatments. Furthermore, metformin, which targets mitochondrial metabolism, also exerts epigenetic effects by activating AMPK, a regulator of both metabolism and chromatin structure [84]. Therefore, combining metformin with DNA methyltransferase inhibitors or HDAC inhibitors could simultaneously address the metabolic and epigenetic vulnerabilities of hepatoblastoma. Studies that combine FASN inhibitors with HDAC inhibitors have also reported enhanced antitumor activity, suggesting that targeting both lipid metabolism and chromatin structure may yield superior therapeutic outcomes [85]. Although these combination strategies are still largely in the experimental phase, they represent a promising direction for future therapies, particularly for patients with recurrent or treatment-resistant disease (Table 2).

### 6.4. Limitations and Future Directions: Clinical Outcomes and Ongoing Trials

While the use of metabolic and epigenetic inhibitors in hepatoblastoma is still in its early stages, promising results from preclinical studies have prompted the initiation of clinical trials. Several phase I/II trials are currently investigating these metabolic and epigenetic agents in pediatric solid tumors, including hepatoblastoma. For example, DNA methyltransferase inhibitors and HDAC inhibitors are being tested in combination with standard chemotherapy regimens to determine whether these epigenetic agents can enhance the efficacy of conventional treatments [80,86]. Additionally, metformin is being evaluated for its role in inhibiting mitochondrial metabolism and as an adjunct to chemotherapy [84]. Early-phase clinical trials have reported encouraging outcomes in reducing tumor burden, and further studies are ongoing to validate its effectiveness in hepatoblastoma. FASN inhibitors and other metabolic agents, although still in the early phases of clinical evaluation, show promise in preclinical models of hepatoblastoma [76]. These trials aim to assess not only the efficacy of metabolic inhibitors but also their safety profiles in pediatric populations. While these therapies are not yet widely adopted in clinical practice, they represent a burgeoning area of research. As more clinical trial data become available, the integration of metabolic and epigenetic inhibitors into standard treatment protocols for hepatoblastoma may significantly improve outcomes, particularly for patients with refractory or recurrent disease.

**Table 2:** The outline of therapeutic targets in HB.

HB Biology	Drugs	Targets	Ref.
Metabolic Dysregulation in Hepatoblastoma	2-deoxy-D-glucose	Glycolysis inhibitors	[73]
	orlistat	FASN inhibitors	[75]
	TVB-2640	Lipid synthesis	[76]
	Metformin	OXPHOS inhibitors	[77]
Epigenetic Dysregulation in Hepatoblastoma	5-azacytidine and decitabine	DNMTis	[79,80,81]
	vorinostat and panobinostat	HDACis	[87]
	miRNA mimics or antagomiRs (anti-miRNA agents)	miRNAs	[63]
Combination Therapies	/	Combination of glycolytic inhibitors with histone modifiers	[83]
	metformin	Mitochondrial metabolism and epigenetic	[84]
	/	Combination FASN inhibitors with HDAC inhibitors	[85]

## 7. Conclusions and Summary

Hepatoblastoma, the most common pediatric liver malignancy, presents unique biological challenges that necessitate innovative therapeutic approaches beyond conventional surgery and chemotherapy. Advances in our understanding of metabolic reprogramming and epigenetic dysregulation have uncovered novel vulnerabilities in hepatoblastoma that can be targeted for therapeutic benefit. Key metabolic pathways, such as aerobic glycolysis, fatty acid synthesis, and oxidative phosphorylation, have emerged as promising intervention targets. Simultaneously, epigenetic modifications, including DNA methylation and histone acetylation, provide opportunities for reprogramming aberrant gene expression in tumor cells.

Despite these advancements, the development of specific inhibitors for pediatric cancers like hepatoblastoma is hindered by challenges such as tumor heterogeneity and potential resistance mechanisms. While combination therapies targeting both metabolic and epigenetic pathways have demonstrated preclinical promise, their clinical translation necessitates further investigation into safety, efficacy, and long-term outcomes. Looking ahead, precision medicine approaches that leverage biomarkers to personalize treatment, along with the integration of immunometabolism into existing therapeutic strategies, could significantly enhance outcomes for hepatoblastoma patients.

Future research must also focus on addressing the epigenetic plasticity of hepatoblastoma to prevent resistance and improve treatment durability. The long-term safety of these therapies, particularly in young children, remains a critical concern that will require careful consideration.

This review highlights recent progress in identifying therapeutic targets in hepatoblastoma, emphasizing the metabolic and epigenetic mechanisms driving tumor growth. Key metabolic targets, including glycolysis and fatty acid synthesis, along with epigenetic regulators such as DNA methyltransferases and histone deacetylases, present new avenues for treatment. Combination therapies that integrate both metabolic and epigenetic inhibitors show potential for enhanced antitumor effects. However, significant challenges, including tumor heterogeneity, resistance mechanisms, and the scarcity of pediatric-specific therapies, remain barriers to clinical translation. Future research should focus on precision medicine, immunometabolism, and overcoming epigenetic resistance, while ensuring the development of safe and effective treatments tailored to the unique biology of hepatoblastoma in pediatric patients. By targeting these emerging pathways, the field of hepatoblastoma treatment could shift towards more effective, personalized, and less toxic therapeutic options, ultimately improving outcomes for children diagnosed with this rare but aggressive cancer.

## Figures and Tables

**Table 1:** The summary of risk factors to hepatoblastoma.

Factors	In Details	Reference
Prematurity and Low Birth Weight	Children born preterm or with a birth weight under 1500 g	[4]
Familial Adenomatous Polyposis	Mutations in the APC gene	[5]
Beckwith–Wiedemann Syndrome	This overgrowth disorder is frequently linked to the abnormal regulation of the imprinted IGF2 gene.	[6]
Trisomy 18 and Trisomy 21	Children with these chromosomal abnormalities have an increased predisposition to liver tumors	[7,8]
Li–Fraumeni Syndrome	TP53 mutations	[9]
